# How practice setting affects family physicians’ views on genetic screening: a qualitative study

**DOI:** 10.1186/s12875-021-01492-y

**Published:** 2021-07-01

**Authors:** Rose Wai-Yee Fok, Cheryl Siow Bin Ong, Désirée Lie, Diana Ishak, Si Ming Fung, Wern Ee Tang, Shirley Sun, Helen Smith, Joanne Yuen Yie Ngeow

**Affiliations:** 1 Cancer Genetics Service, Division of Medical Oncology, National Cancer Centre Singapore, Singapore, Singapore; 2 Sociology, School of Social Sciences and Lee Kong Chian School of Medicine, Nanyang Technological University, Singapore, Singapore; 3 Signature Programme in Health Services and Systems Research, Duke-NUS Medical School, Singapore, Singapore; 4 National Healthcare Group Polyclinics, Singapore, Singapore; 5 Lee Kong Chian School of Medicine, Nanyang Technological University, 11 Mandalay Road, Singapore, 308282 Singapore; 6 Oncology Academic Clinical Program, Duke NUS Medical School, National University Singapore, Singapore, Singapore

**Keywords:** Genetic screening, Primary care, Family physicians, Attitudes, Private, Public

## Abstract

**Background:**

Genetic screening (GS), defined as the clinical testing of a population to identify asymptomatic individuals with the aim of providing those identified as high risk with prevention, early treatment, or reproductive options. Genetic screening (GS) improves patient outcomes and is accessible to the community. Family physicians (FPs) are ideally placed to offer GS. There is a need for FPs to adopt GS to address anticipated genetic specialist shortages.

**Objective:**

To explore FP attitudes, perceived roles, motivators and barriers, towards GS; and explore similarities and differences between private and public sector FPs.

**Methods:**

We developed a semi-structured interview guide using existing literature. We interviewed private and public sector FPs recruited by purposive, convenience and snowballing strategies, by telephone or video to theme saturation. All sessions were audio-recorded, transcribed and coded for themes by two independent researchers with an adjudicator.

**Results:**

Thirty FPs were interviewed (15 private, 15 public). Theme saturation was reached for each group. A total of 12 themes (6 common, 3 from private-practice participants, 3 public-employed participants) emerged. Six common major themes emerged: personal lack of training and experience, roles and relevance of GS to family medicine, reluctance and resistance to adding GS to practice, FP motivations for adoption, patient factors as barrier, and potential solutions. Three themes (all facilitators) were unique to the private group: strong rapport with patients, high practice autonomy, and high patient literacy. Three themes (all barriers) were unique to the public group: lack of control, patients’ lower socioeconomic status, and rigid administrative infrastructure.

**Conclusion:**

FPs are motivated to incorporate GS but need support for implementation. Policy-makers should consider the practice setting when introducing new screening functions. Strategies to change FP behaviours should be sensitive to their sense of autonomy, and the external factors (either as facilitators or as barriers) shaping FP practices in a given clinical setting.

**Supplementary Information:**

The online version contains supplementary material available at 10.1186/s12875-021-01492-y.

## Background

Genetic screening (GS), is defined as the clinical testing of a population to identify asymptomatic individuals with the aim of providing those identified as high risk with prevention, early treatment, or reproductive options [1, 2]. This includes presymptomatic genetic testing to evaluate a healthy person to determine if the person will develop a condition (e.g. Huntington’s disease). Another is predisposition genetic testing where it informs individuals of an increased or decreased risk of developing a condition (e.g. *BRCA1/BRCA2*)*.* The third type of testing is intended to help couples make reproductive decisions (e.g.Thalassaemia) [3, 4].

GS is the foundation of precision medicine, proven to improve patient outcomes [5, 6], and already applied in oncology, pharmacogenetics and prenatal diagnostics. Recently, GS has been used to improve chronic disease care such as diabetes [7]. Given limited specialist resources to meet the rising demand for genetic services [8], there is a role for FPs to adopt and deliver GS in primary care [9, 10].

Family physicians (FPs) provide the bulk of primary care services [11] in most health systems and are ideally suited to offer disease screening because they care for patients across the lifespan [12]. Their guiding principles of continuity of care, with coverage of urgent, chronic, preventive and end-of-life care [13] place them in a unique position to counsel and advise patients on long-term and heritable health conditions. FPs are trusted by patients to gauge the best timing for such screening and to advise patient’s relatives, an advantage which may not be shared with secondary and tertiary providers who are providing more episodic care [13, 14].

Studies have suggested that FPs see themselves as pivotal in maintaining wellness and health as a part of their professional identity and job satisfaction [15]. Preventive healthcare such as cancer and chronic disease screening are already part of most FPs practice scope and skillset. A systematic review reported that FPs perceived genetics as being important and valued the promotion of practice guidelines, risk assessment tools, tailored education and system-level strategies to assist them in adoption [16].

Potential roles of FPs in GS include taking family history information, discussing genetic risk, appropriate referral, obtaining genetic tests and informing individuals of their results and significance [17]. Communicating genomic risk, adopting a shared decision making approach, may soon become a responsibility of primary care [18]. FPs also identified their roles in providing emotional support, teaching breast self-examination and discussing need for screening [19]. Concurrently, patients expect their FPs to play a role in risk identification, genetics referral and ongoing role after receiving the genetic test results [20]. However there are multiple barriers for this expanded role, which include limited knowledge, lack of confidence, deficiency of training in genetic counselling, shortage of specialist community support, inadequate educational programmes and referral guidelines [21]. Little is known about FPs’ readiness and willingness to integrate GS into their practices, or their perception of who in the health system should take responsibility for this function.

Primary care is defined as a whole-society approach to health and well-being for individuals, families and communities [22]. In Singapore, primary care is delivered by registered family physicians with post-graduate academic training and qualifications that meet nationally-defined accreditation standards [23, 24]. A small proportion of older private-practice general practitioners were grandfathered in as registered FPs based on their longevity of community practice. The private sector is a market-driven fee-for-service model. The government-subsidised public system provides care to all citizens as a safety net. Together, both private and public sectors provide all primary care services.

70 to 80% of the population receive primary care from about 1,500 private practitioners who provide care in solo or small group practices. The remaining 20 to 30% receive care from over 500 FPs working in 20 publicly-funded practices, each of which also offers care from nurses, pharmacists and allied health professionals [25].

The willingness and ability of FPs to include GS as part of their routine scope of practice may be influenced by practice setting. Differences in practice patterns have been observed between FPs in the private and public sectors, who may differ in their ability to control their schedules, patient flow, reimbursement, practice structure and the application of clinical practice guidelines [26]. An understanding of potential differences in attitude toward GS will allow targeted strategies to enhance practice adoption.

We therefore conducted an exploratory qualitative study to examine the attitudes and perspectives of both private-practice and publicly-employed FPs to investigate the potential to involve them in community GS. Our goal was to identify motivators and barriers and to identify effective approaches to enhance genetic services in the community.

## Methods

### Conceptual framework

We identified self-determination theory [27, 28] as a useful framework for our study. This conceptual framework proposes that people are motivated to change behaviour when they perceive greater autonomy and competence and feel valued and respected. Physicians generally display high levels of intrinsic motivation which drive them to pursue goals that bring job satisfaction, fulfilment and commitment. Physicians may also be driven by external motivators. Studies suggest that practice behaviours can vary among physicians and are not necessarily purely influenced by scientific evidence or knowledge [29, 30]. The self-determination framework allows us to examine factors that motivate or hinder family physicians in the choice to incorporate a new emerging screening service in patient care.

### Study design

A qualitative methodology was considered more appropriate than a survey because it allows open-ended exploration of factors as yet unknown or unreported. We chose in-depth interviews (IDIs) over focus groups to delve deeper into personal views and to allow expression of both positive and negative perceptions which FPs may not wish to share with their peers [31].

A semi-structured interview guide was developed from a review of existing literature and discussion [16, 32–34]. This guide was refined by a clinical geneticist (JN), practising FP (RF), FP educator/ researcher (DL), sociologist (SS), and qualitative researcher (SMF). The interview guide was pilot-tested with two practising FPs, resulting in minor revisions (Table 1). Probes, prompts and follow-up questions were added.

**Table 1:** Topic guide

Key Questions	Probes
**Attitudes and perception** 1. What do you understand about GS^a^? How relevant is GS to your patient population?	• How have you learned about GS? • How prepared are you to counsel patients or discuss GS? (If unprepared, explain why not)
**Experience** 2. What is a typical day in your clinic? What guides you to screen patients in your practice?	• What GS have you recommended? • (If with experience) Please share your patient experience with GS
**Adoption in clinical practice** 3. What challenges do you face, or anticipate facing, with adopting GS in your clinical practice?	• What do you think would motivate you to prepare for these challenges? • What are the pros/cons of GS for you and your patients?
**Informational and infrastructural needs** 4. How would you like to be supported when using/adopting GS in your clinical practice?	• What information about GS would be helpful? • How would you like to learn more about GS?

^a^*GS* Genetic screening

### Study setting

Our study was conducted in Singapore, a sovereign island city-state of 5.6 million comprising a diverse mix of Asian ethnicities (Chinese, Malay, Indian, other).

We used a combination of purposive, convenience and snowballing sampling strategies via emails and flyers [35] to recruit FPs from different practice settings with diverse qualifications, seniority and work experience. Our recruitment materials stated our interest in views about GS, to select for information-rich sources. We also sent personal invitations via clinic directors. Private FPs were recruited from 7 solo and 4 group private practices. Public FPs were recruited from 8 public practices located in the east, west and central regions. We aimed for equal representation of views from private and public FPs. FPs received emails with telephone follow-up to confirm participation.

The primary interviewer was an experienced female FP (RF) who conducted 83% of interviews. Her initial 6 transcripts were reviewed by the research team which provided feedback. The remaining 17% of interviews were conducted by the qualitative researcher (SMF) and sociology research assistant (CO) with training from RF.

### Data collection

We obtained written informed consent before each interview. Before each session, participants completed an anonymised demographic survey. In-person interviews, lasting 45 to 60 min, were conducted in private rooms convenient to the participants. In April 2020, we converted in-person to a virtual platform using video-conferencing Zoom® software, with audio recording only for transcripts, due to the Covid-19 pandemic. Based on existing studies [36–39], we considered data gathered by video to be equivalent to that from in-person interviews. Only the participants and interviewers were present in both in-person and video-conferencing interviews. Confidentiality was maintained by de-identifying the transcripts. The recordings, transcripts, coding, field notes and coders’ reflexive notes, taken during the interviews, were organized in secure electronic archives, to establish a clear audit trail [40].

Each participant was remunerated with Singapore $40 dollars for their time. All sessions were audio-recorded and transcribed verbatim. The interviews continued until data saturation was reached when no new themes emerged for either FP group [41].

### Data analysis

We applied the Braun and Clarke six-stage thematic analysis framework [42] for analysis. The primary coders (RF, CO) read through the transcripts independently to familiarize themselves with the data. They conducted individual line-by-line coding to generate a list of initial codes. They then met and organized their separate data into meaningful groups to create a codebook, and collated the collective data into a broader level of themes. The themes were reviewed, refined and defined, then named. Lastly, they created a table of the themes generated with representative quotes. The process was repeated for both, and for each FP group to identify common and unique themes. Participant demographics were summarised using descriptive statistics.

## Results

### Participants

Out of the 40 FPs approached, six did not respond and four declined participation due to self-reported lack of knowledge. Thirty FPs from 15 private-practices (15 FPs) and eight public practices (15 FPs) were interviewed from March to October 2020. The IDIs were conducted face-to-face for the first seven FPs, and by video-conferencing for the remaining 23 interviews. No repeat interviews were needed.

Majority of the participants were Chinese (Table 2). Public FPs were significantly younger (mean 36 vs 45 years). 86% of public vs 47% of private FPs had less than 16-years of work experience. A greater number of public FPs have higher qualifications compared to private FPs. Both groups reported seeing more than 40 patients daily.

**Table 2:** Participant demographics

**Characteristic**	**Private-practice, 15 (%)**	**Public-employed, 15 (%)**
**Gender**		
Male	9 (60%)	7 (47%)
Female	6 (40%)	8 (53%)
**Ethnicity**		
Chinese	12 (80%)	13 (87%)
Indian	2 (13%)	0 (0%)
Others	1 (7%)	2 (13%)
**Practice experience (years)**		
5–10	5 (33%)	8 (53%)
11–15	2 (14%)	5 (33%)
16–20	3 (20%)	1 (7%)
> 20	5 (33%)	1 (7%)
**Age (years)**		
30–39	7 (47%)	12 (80%)
40–49	3 (20%)	2 (13%)
> 49	5 (33%)	1 (7%)
**Average number of patients seen per day**		
< 20	0 (0%)	1 (7%)
20–40	5 (33%)	4 (26%)
> 40	10 (67%)	10 (67%)
**Qualifications**		
Master FM^a^ training	10 (67%)	15 (100%)
Advanced FM training	4 (26%)	5 (33%)

^a^*FM* Family medicine

The two primary coders created the initial code book using 6 transcripts on an Excel spreadsheet. After independent coding, 4 common major themes were identified. 18 additional interviews were conducted which yield twelve other themes. Saturation was confirmed after conducting an additional 6 interviews. After discussion, consolidation, and adjudication by the adjudicator (DL), 6 common major themes remained. Coding of the private and public FP transcripts yielded 3 major themes each, with saturation reached and confirmed after 15 and 15 interviews, respectively (Additional file 1).

### Common themes

#### Personal lack of training and experience

Although most participants recognized the benefits of GS, their perceived level of confidence to conduct GS was low, attributed to a lack of training and knowledge. FPs reported:

*“It is not in our training, not covered in medical school. In my post-grad training, I have never been educated on this.” Public14*

*“Not much is known (to FPs) about genetic screening, the facilities available, where to go for testing, knowledge about the diseases, and counselling. You need all those factors before you can manage patients.” Private12*

Many participants reported few encounters involving genetic issues, which contributed to their perceived lack of experience.

*“I don’t think we would have the experience. If you see these cases once every few months, then…we cannot offer the same standard of care as a specialist.” Public14*

#### Roles and relevance of genetic screening

Participants perceived usefulness of GS to conditions commonly seen in their practice, for example:

*“(In)…familial hyperlipidaemia,…there is a huge implication on the patient as well as their family members but right now we cannot confirm that (without genetic testing)…Other things would be HLA testing,…thalassemia and Down’s syndrome…” Public10*

Participants generally perceived themselves to be well-positioned to offer GS in line with the ethos of family medicine and the geographic proximity of patients and families to their practices.

*“…the principles of family medicine are to…(provide)…comprehensive care,…so definitely genetic testing should be included…we know the patients…best,…and sometimes we have relationships with their family members.” Public11*

*“I think that the FP would be a very good person for these kinds of genetic conditions. People that come to see me are living nearby. So even if their family does not regularly see me, they (live) nearby and can always bring the family if the need arises.” Private14*

However, they expressed ambivalence about their current roles and competency to practise GS and the need to refer to specialists. They were cognizant that they needed to become competent in order to adopt GS.

*“specialists should be the ones who do the (GS)…what we in primary care can do is…to be aware, or need to be educated…so that when patient come and ask us, we can actually advise.” Public3*

#### Reluctance and resistance

One viewpoint was that genetic testing was a new arena which may “open a can of worms” for which FPs were ill-prepared to manage.

*“If the (test result) is positive it is like opening a can of worms. (The patient) might not be able to handle it and that is one of the reasons a physician might not want to take on this…” Public15*

Some perceived that offering GS was not core to their scope of practice because of the rarity of genetic conditions.

*“I rarely recommend, because it’s not the standard of care, not a compulsory thing …maybe once in a while, I’ll see a neurofibromatosis patient and then I’ll refer them but that’s really rare.” Public5*

*“I think there is limited scope for GS in primary care in terms of the spectrum of patients and the main chronic diseases that we manage…there may be a certain role for GS in a very, very small group.” Public14*

#### FP motivations for adoption of GS

FPs interviewed expressed the view that GS may be of interest unique to some but not all FPs and could be offered as a special service. These quotes reflect this opinion:

*“So it depends on the interests also of the doctor, so some want to go further, draw genograms…and then advise the family.” Private11*

*“Best is to have a dedicated clinic just to run this kind of (GS).” Public 12*

Other motivators included affordable costs and national consensus. These views were expressed in these quotes:

*“…if the cost is okay,…the test is reliable and…able to provide us with more information to better the preventative health of the patient, then it will be good.” Public13*

*“If there are clear guidelines from the government and they can come up with some financial support that would help. Also if all health practitioners are on board, including the specialists, then the patients are more likely to be convinced to do this genetic testing” Private14*

#### Patient factors as barrier

Participants identified negative patient attitudes as a potential barrier. They were concerned that offering GS would add an emotional and psychological burden over and above the current screening procedures. However they also perceived that education may overcome these barriers. These quotes express their fears:

*“I have faced difficulties getting patients to even go for their routine screening...patients are not keen to test as they don’t want to worry or they just want to live their life happily.” Public9*

*“…you are making a person worry over a onset of a disease that has not taken place yet…by giving a label, you are making a person into a patient…ethically, do we wish to put a kind of mental burden onto a person?” Private11*

#### Potential solutions for implementing GS

Participants indicated that GS adoption would be greater if Continuing Medical Education (CME) and other educational and systems support were offered. Views about education, electronic reminders and easy referral to geneticists include the following:

*“ongoing information support would be useful…CME talks, brochures, or pamphlets…to remind FPs.” Public15*

*“(We need) guidelines…followed up with interactive teaching, workshops and lectures.” Private12*

*“…a template in-built into our IT system…where we can assess risk based on family history…help us identify patients who may benefit from (GS).” Public8*

FPs advocated for sufficient consultation time, care coordination, referrals pathways, common consensus and a right care model with financial subsidies.

*“what we lack is coordination…coordination takes time, effort, expertise and relationships…” Private5*

FPs perceived that raising the status of GS would build confidence in adopting GS.

*“…if you raise the status and there is formal recognition, then I think it’s more appealing…FPs can feel more supported (and) more confident in offering this.” Public7*

### Unique themes: private-practice

All three major themes for the private-group were facilitators of GS adoption.

#### Strong longitudinal patient relationship

Private-practice FPs’ access to and longitudinal relationship with patients and family members allow them to build rapport and enhance collection of family history to understand their genetic predispositions and offer GS as appropriate.

*“the (private) FP, plays a huge part because we have the privilege of…see(ing) patients from cradle to grave.” Private13*

#### Practice autonomy

Private FPs expressed that they perceive autonomy to advocate for GS. This view was expressed in the quote below:

*“If (GS) is easy to utilise, it would be a factor in whether we use the test, besides the cost and demand…the ease of use…would be a major factor.” Private12*

#### Higher patient literacy

Private FPs perceived that their patients had high health literacy and may be more accepting of GS. One FP reported:

*“We are beginning to see young patients less than forty (years old), they are more educated…more willing to test…often driven by fear. So on one hand they want to test but on the other hand they are also worried about the results. I need to be quite careful in counselling them.” Private2*

### Unique themes: public-employed

All 3 major themes for the public group were obstacles to GS adoption.

#### Lack of control

Public FPs perceived lack of control over their schedules and practice structure as a barrier. For example,

*“we still need to see acute cases, chronic disease management which is our bread and butter…There’re a lot of healthcare initiatives and projects that primary care is involved in. With all these, time constraint is the big challenge” Public11*

FPs also reported less continuity of care in the public sector. For example, one said:

*“….in the public sector, many patients don’t have a fixed doctor…If you see a different doctor each time, it’s very hard (for FPs to offer GS).” Public9*

#### Lower patient socioeconomic status and literacy

Public FPs perceived their patients to have limited literacy and low socioeconomic status making it harder for them to persuade their patients to adopt GS.

*“If the FP assesses that the patient is not capable or does not have the intellectual understanding of what genetic testing is, they might not want to offer GS...(the) FP might not want to deal with, or explain it to the patient.” Public15*

#### Rigid administrative infrastructure

Public sector FPs perceived greater bureaucratic and administrative obstacles to adopting GS in their practices. Quotes reflecting this opinion include the following:

*“I think (GS) is quite a new field and senior administrators are not aware. That’s the reason why it’s not being pushed out to the public clinics.” Public1*

*“The leaders…the policy makers…We need their support…without them, we cannot do anything.” Public6*

## Discussion

### Summary of current findings

We conducted an IDI study to explore opinions about adopting GS among private and public sector FPs. This study identified a total of 12 themes (6 common, 3 private-practice, 3 public-employed) arising from different practice settings which are linked to intrinsic and extrinsic motivators. The six common themes were: personal lack of training and experience, roles and relevance of GS to family medicine, reluctance and resistance to adding GS to practice, FP motivations for adoption, patient factors as barrier, and potential solutions. All three themes unique to the private group represented positive incentives to screen (strong rapport with patients, high practice autonomy, and high patient literacy) while all three unique to the public group were barriers (lack of control, patients’ lower socioeconomic status, and rigid administrative infrastructure).

**Fig. 1:**
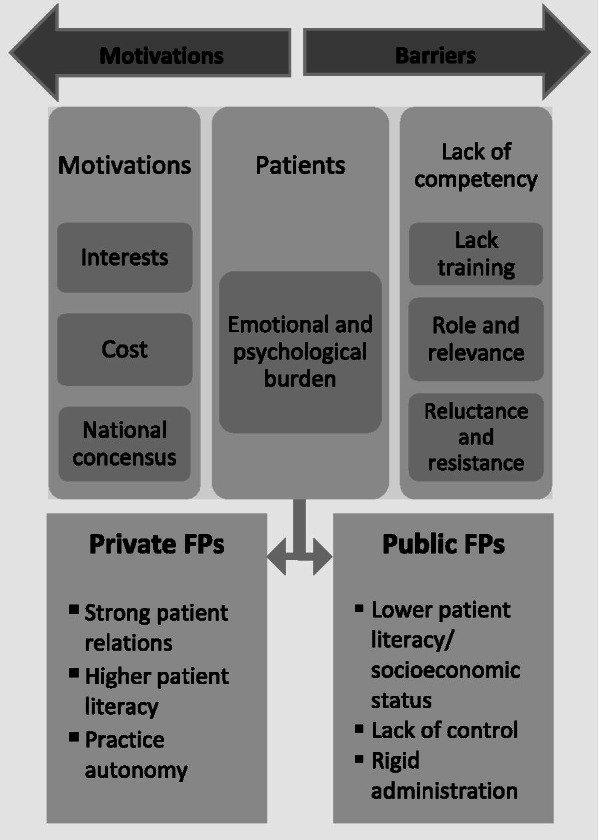
Self-determination framework for understanding private and public family physicians’ attitudes to adopting genetic screening

Our study is unique in adding new information about differences in perception and motivation between private and public sector FPs in keeping with the self-determination framework. Private-practice FPs perceive high autonomy and control over their practices and are open to expanding their scope. This contrasts with public sector FPs who perceive less autonomy over decision-making and practice policy, and are more likely to be extrinsically motivated by directives or policy guidance from their superiors. Both groups express responsiveness to incentives but the nature of those incentives differ between the groups. Patient literacy and continuity of care were important external motivating factors that differed between the two groups. Figure 1 illustrates both the continuum and dichotomy of motivators and barriers between the two groups.

It is notable that we found more barriers than motivators among the younger public FPs (who graduated more recently, possessed higher qualifications) with less practice experience, while older private FPs with more years of practice expressed a more positive attitude toward GS. This dichotomy expressed through the unique themes underscores the power of practice environment, patient profile and systemic support on attitudes toward adopting GS.

### Comparison with existing literature

Two (lack of experience/ training, and reluctance/ resistance) of six major themes common to both groups reflect the ambivalence of FPs to embrace an enhanced role in GS. They were cautious about their ability to gather accurate information due to possible errors in patient recollection or knowledge, resulting in subsequent risk misinforming patients as well as competing demands of other services, all findings consistent with literature [12, 43]. The five perceived barriers we identified have been previously reported and include low disease prevalence, high patient cost, own lack of knowledge, experience and confidence [12, 16, 44, 45]; time limitations and lack of referral guidelines [46, 47]. For example, FPs in Canada considered GS outside their practice scope [48]. Previous research found that FPs acknowledged that GS is a valuable tool in healthcare but required more evidence that incorporating it into primary care will improve patient outcomes [12].

In our study, patient resistance (negative attitudes, lower socioeconomic and healthcare literacy) were additional factors noted by FPs. Our findings correspond with previous work that patients relate information about genetic risk from their personal experience of the disease and beliefs about inheritance rather than scientific explanations [49]. Supplementing prior studies, our participants advocated for more FP education to address patient misconceptions [50]. However, despite their reservations, FPs in our study demonstrated empathy and concern for their patients. Our themes reflected their consideration of patients’ viewpoints, both negative (e.g. risk of emotional and psychological harm) and positive (e.g. benefits of risk reduction strategies or family planning options). A study concur that GS could harm patients by causing anxiety, for example, when a test result is positive but treatment was unavailable, or when there was uncertainty when the disease would manifest [12].

FPs considered their own and their colleagues’ unique interests in GS as a pathway to offer specialized GS clinics in the community. A study showed that facilitators of GS were positive attitudes of FPs towards genetics, willingness to participate in discussions upon patient initiation, and intention to engage in genetic education [51]. It has been shown that through increasing familiarity through participatory learning, FPs demonstrated higher interest towards GS, greater confidence and likelihood of recommending testing to interested patients [52].

FPs have been open to integrating GS into primary care practice since the introduction of GS over 20 years ago [17], citing inadequate education and preparation as limiting factors. FPs in our study perceived that their medical training did not prepare them adequately for clinical adoption of GS. They advocated for more genetics content in medical school, residency and CME activities, suggestions which concur with existing literature [53, 54].

In the intervening time, some progress has been made to address the physician knowledge gap, attitudes and communication-related behaviours regarding GS [55]. However education per se is unlikely to change attitudes or practice significantly if those changes do not fit into their current roles or benefit their practice. Despite formulation of genetic curriculum in undergraduate and postgraduate training [56], FPs’ reluctance to engage with clinical genetics persisted, suggesting that knowledge deficit models do not fully explain FP behaviour [43].

### Study strength and limitations

Little is known globally of how different practice settings might impact uptake of new practice guidelines such as GS. Our study extended this understanding. Strengths of our study include broad sampling and representation from a diverse group of community FPs, theme saturation for both practice groups, and a robust coding process yielding important comparative data. We had access to two important settings in which most FPs practice. Importantly, our researchers represented primary care, genetics, sociology and public health perspectives. Our study may be limited by a primarily urban healthcare structure not shared by all primary care systems. However the differences in practice and functions between our private and public FPs reflect core differences seen across many global systems ranging from completely public to mainly private, including hybrid systems. Another limitation is the influence of selection bias as FPs who participated had some prior knowledge of GS. Despite this, however, majority of our interviewees reported not being prepared to educate patients on GS or to manage surveillance for high-risk patients.

### Clinical implications

Our findings have implications for future policy to implement GS and other new screening guidelines. Practice context should be considered. Different educational and infrastructure support strategies respecting individual FP motivations and abilities (self-determination model) [27, 28] are needed. Longitudinal education beginning in medical school, boosted and consolidated during residency training, and CME for practitioners and interdisciplinary/ interprofessional collaboration are foundational to increase physician knowledge and readiness for genomic medicine applications [57, 58]. However, increase in FPs’ perceived knowledge alone may not drive adoption of GS, as busy physicians also need systems-level support to engage in meaningful discussions around genetic conditions [59, 60]. Engaging FPs proactively as part of joint planning of new practice policies is central to future implementation, as patients value the opinion of their healthcare provider [61]. In addition, it remains to be seen whether patients and family members will embrace GS, even if universally offered by their personal FPs [62].

Future research will test the effectiveness of online and in-person educational programs and built-in electronic risk assessment and recommendations for FPs, in promoting GS; and link FP screening behaviours to patient acceptance and utilization of these services.

## Conclusions

FPs are motivated to provide best care for their patients and open to incorporating GS but need education and support for implementation. Policy makers should consider practice setting when introducing new clinical functions. Strategies to motivate FPs should be sensitive to their sense of autonomy and control, encourage relatedness to colleagues and patients and respect for their competence.

## Supplementary Information

**Additional file 1.** Common and unique themes with representative quotes.

## Data Availability

The datasets analysed during the current study are available from the corresponding author on reasonable request.

## Abbreviations

CME: Continuing medical education
FP: Family physician
GS: Genetic screening
HLA: Human leukocyte antigen
IDI: In-depth interview
IT: Information technology
