# Supplementary material for: How practice setting affects family physicians’ views on genetic screening: a qualitative study
Source: BMC Fam Pract. 2021 Jul 1;22:141. doi: 10.1186/s12875-021-01492-y (PMC8247620; doi:10.1186/s12875-021-01492-y)
Supplement: Supplementary file 1 — Additional file 1. Common and unique themes with representative quotes. [file 12875_2021_1492_MOESM1_ESM.docx]

**Additional file 1: Common and unique themes with representative quotes**

| **Common Themes** | **Representative quotes, by practice setting** |
| --- | --- |
| Personal lack of training and experience | - *“Not prepared at all...I'm not sure how to counsel someone (with) a genetic test result.” Public9* - *“doctors are not aware,...(how to) give the right advice...how to interpret and advise (patients) what to do.” Public2* |
| Roles and relevance of GS | - *“if your parents... had a recent heart attack then some patients would want to know...(if) it is preventable, (or) give them (a drug) to reduce their risks, (then) it is indicated, (and) actionable” Private7* - *“my role (is) more in educating them…what it's about and what relevance it has in their lives.” Private9* |
| Reluctance and resistance | - *“… I think knowledge is lacking and … the general interest is also quite low in this topic…usually the mindset of FPs with regards to GS is it's in the specialists' domain.” Public7* - *“(Adoption of) GS (is) more than just equipping the primary care physicians with the knowledge but also whether the infrastructure can be there to support.” Public10* - *“We (FPs) need to be well versed...in the law, implications and insurance, before I would even consider GS” Private3* - *“I (FP) don't want to manage that kind of situation where…, genetic negative but disease still come out” Public* |
| FP motivations for adoption of GS | - *“Motivate me would be my patients, if I had more patients with this problem, that would be the motivation to overcome the challenges, to learn more, to get more information, better counselling. But if it's something that's not relevant to my practice, there's no motivation to do it.” Private12* |
| Patient factors as barrier | - *“A lot of times, (patients) don't want to divulge certain family history in the fear...whether it will affect their employment, (and) insurance later. Private9* - *“…I think a lot of the elderly feels that cancer is quite a taboo.” Private2* |
| Potential solutions for implementing GS | - *“useful and important government policies that will support… especially the financial burdens that the patients might have for taking up some of these tests.” Public15* - *“…also educating the public to let them know that, ... what kind of genetic disorders are common in Singapore and what are the risk factors and actually it's not just about tertiary care,...(but what) primary care can do... for you.”Public11* |
| **Unique Themes: Private-practice** | **Representative quotes** |
| Strong longitudinal relationship | - *“…we can engage the patient in further discussion and ask about the whole family…we can build upon the rapport to... further the course and improve outcome.” Private3* |
| Practice autonomy | - *“This is demand versus economic viability… if it's very much in demand then even if it's expensive, we will give serious consideration as to how we can overcome the challenge and provide it.” Private1* |
| Higher patient literacy | - *“if you go into a more affluent area then they will be more proactive, …testing themselves and then doing something preventative to prevent themselves from getting the disease.” Private7* - *“there is quite a sizable population of… thalassemia patients in Singapore, so some of them are interested to check whether they do have thalassemia. Especially the women who in the childbearing age who want to know if they carry the gene as it is relevant for their future pregnancy.” Private15* |
| **Unique Themes: Public-employed** | **Representative quotes** |
| Lack of control | - *“it will be the time constraints so even if I have the skills, I know how to explain, I might not have the time to do so.” Public9* - *“if I'm the only one doing it with no HQ support, no cannot. I'll be setting a bad example for my clinic.” Public8* |
| Lower socioeconomic status and literacy | - *“for the elderly, it is both ... economic cost and … literacy. And for those who are working, they might be literate but cost is also still an issue.” Public3* |
| Rigid administrative infrastructure | - *“I work in a public sector, … we are linked up quite closely with the specialist colleagues... (and) it is very accessible to us to refer on (to specialists) then there is no reason why we wouldn’t (refer on) rather than take on the load ourselves.”Public14* - *“I work in the public setting, so everything I do must be approved by the board and must follow the protocols.”Public2* - *“Because in our setting, we don't have a huge group of patients with this kind of genetic predisposition…So the cost-benefit may not quite justify the setting up of the separate clinic…unless…the prevalence is quite high and it's worth doing it.” Public12* |

GS: genetic screening FP: family physicians HQ: headquarters
